# Naturally acquired immunity to *Plasmodium pitheci* in Bornean orangutans (*Pongo pygmaeus*)

**DOI:** 10.1017/S0031182024000155

**Published:** 2024-04

**Authors:** Karmele Llano Sánchez, John Kevin Baird, Aileen Nielsen, Andini Nurillah, Fitria Agustina, Komara, Fina Fadilah, Wendi Prameswari, Raden Taufiq Purna Nugraha, Sugiyono Saputra, Arif Nurkanto, Anik Budhi Dharmayanthi, Rahadian Pratama, Indra Exploitasia, Alex D. Greenwood

**Affiliations:** 1 IAR Indonesia Foundation, Yayasan Inisiasi Alam Rehabilitasi Indonesia (YIARI), Sinarwangi, Bogor, West Java, Indonesia; 2 International Animal Rescue, Uckfield, UK; 3 School of Veterinary Medicine, Freie Universität, Berlin, Germany; 4 Oxford University Clinical Research Unit-Indonesia, Faculty of Medicine, Universitas Indonesia, Jakarta, Indonesia; 5 Centre for Tropical Medicine and Global Health, Nuffield Department of Medicine, University of Oxford, Oxford, UK; 6 Center for Law and Economics, ETH Zurich, Zurich, Switzerland; 7 Research Center for Applied Zoology, National Research and Innovation Agency (BRIN), Jakarta, Indonesia; 8 Research Center for Biosystematics and Evolution, National Research and Innovation Agency (BRIN), Jakarta, Indonesia; 9 Center for Biomedical Research, Research Organization for Health, National Research and Innovation Agency (BRIN), Jakarta, Indonesia; 10 Biodiversity Conservation Directorate of the General Director of Natural Resources and Ecosystem Conservation, Ministry of Environment and Forestry of the Republic of Indonesia, Jakarta, Indonesia; 11 Department of Wildlife Diseases, Leibniz Institute for Zoo and Wildlife Research, Berlin, Germany

**Keywords:** malaria, naturally acquired immunity, One Health-One Welfare, orangutan, orangutan conservation, *Plasmodium pitheci*, veterinary parasitology

## Abstract

Naturally acquired immunity to the different types of malaria in humans occurs in areas of endemic transmission and results in asymptomatic infection of peripheral blood. The current study examined the possibility of naturally acquired immunity in Bornean orangutans, *Pongo pygmaeus*, exposed to endemic *Plasmodium pitheci* malaria. A total of 2140 peripheral blood samples were collected between January 2017 and December 2022 from a cohort of 135 orangutans housed at a natural forested Rescue and Rehabilitation Centre in West Kalimantan, Indonesia. Each individual was observed for an average of 4.3 years during the study period. Blood samples were examined by microscopy and polymerase chain reaction for the presence of plasmodial parasites. Infection rates and parasitaemia levels were measured among age groups and all 20 documented clinical malaria cases were reviewed to estimate the incidence of illness and risk ratios among age groups. A case group of all 17 individuals that had experienced clinical malaria and a control group of 34 individuals having an event of >2000 parasites μL^−1^ blood but with no outward or clinical sign of illness were studied. Immature orangutans had higher-grade and more frequent parasitaemia events, but mature individuals were more likely to suffer from clinical malaria than juveniles. The case orangutans having patent clinical malaria were 256 times more likely to have had no parasitaemia event in the prior year relative to asymptomatic control orangutans. The findings are consistent with rapidly acquired immunity to *P. pitheci* illness among orangutans that wanes without re-exposure to the pathogen.

## Graphical abstract

**Figure:**
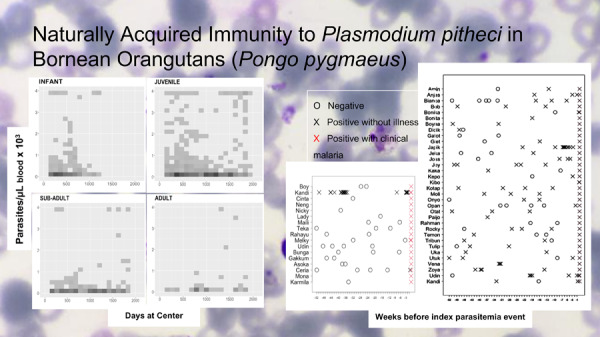

## Introduction

Four species of the genus *Plasmodium* are adapted to humans as their primary intermediate host and cause pathogenic infections (Lindblade *et al*., 2014). The presentation of clinical malaria in human patients ranges from mild and uncomplicated to complicated and severe with variable potential for fatal outcomes (Wickramasinghe and Abdalla, 2000). Active plasmodial infection of the bloodstream may also occur without illness. This asymptomatic malaria is defined as the observed presence of asexual *Plasmodium* spp. parasites in blood (patent parasitaemia) in the absence of fever or any other observable signs of disease (Lindblade *et al*., 2014; World Health Organization, 2015; Sumbele *et al*., 2015; Chen *et al*., 2016; Botwe *et al*., 2017). Chronic asymptomatic malaria may be associated with sub-clinical effects such as anaemia (Owusu-Agyei *et al*., 2001; Lindblade *et al*., 2014; Chen *et al*., 2016; Pava *et al*., 2016). Sexual gametocytes in human blood do not provoke illness. The hepatic pre-patent and latent states of malaria infection also occur without illness but do so naturally (in numbers insufficient to provoke illness) and are not further considered here in the context of immune suppression of the otherwise pathogenic characteristics of patent asexual parasitaemia events.

In some malaria-endemic areas up to 75% of infections of peripheral blood may be asymptomatic and rarely progress to severe disease among most demographic groups (Kinyanjui, 2012; Teun *et al*., 2014). Where this occurs, the highest susceptibility to severe malaria illness occurs in infants, small children and pregnant women, especially the primigravidae (Baird *et al*., 2003; Doolan *et al*., 2009; Guinovart *et al*., 2012; World Health Organization, 2016, 2019). Similarly, immuno-naïve adults suffering from a new infection (Baird *et al*., 1998) (e.g. visitors to malaria-endemic countries from non-endemic areas; Baird *et al*., 2003) are at much higher risk of severe and threatening acute illness of varied syndromes (Kinyanjui, 2012; World Health Organization, 2019). Variably understood factors explain this wide spectrum of clinical disease (Joyner *et al*., 2017). The dominating mitigation of illness with endemic infection is widely accepted as a naturally acquired and non-sterilizing immunity (Guinovart *et al*., 2012; Lindblade *et al*., 2014). Host genetic factors may also influence morbidity, disease severity and mortality of malaria infection in humans (Fortin *et al*., 2002; Botwe *et al*., 2017), but less markedly and commonly than naturally acquired immunity.

Human malaria immunity is typically observed epidemiologically, with striking impacts on the prevalence and density of parasitaemia, and frequency of morbidity and mortality across age groups at the population level. This naturally acquired age-dependent immunity is believed to develop first by creating immunity to clinical disease early in life and later protect from the illness by reducing parasite loads in blood (Doolan *et al*., 2009; Kinyanjui, 2012). However, paradoxically, malaria-naïve adults or those who experienced a prolonged period of non-exposure (e.g. in excess of 2 years) are also at a high risk of suffering from clinical acute malaria (Owusu-Agyei *et al*., 2001; Baird *et al*., 2003; Doolan *et al*., 2009) even with relatively low-grade parasitaemia (Owusu-Agyei *et al*., 2001; Doolan *et al*., 2009). These trends are invariably age-dependent, albeit in varied patterns depending on local character of malaria transmission (Owusu-Agyei *et al*., 2001; Baird *et al*., 2003, 2012; Basri *et al*., 2003; Baird and Snow, 2007; Doolan *et al*., 2009; Barry and Hansen, 2016).

Naturally acquired immunity was long considered a phenomenon restricted to areas of relatively intense exposure, as in sub-Saharan Africa or the island of New Guinea. The relatively recent exploration of malaria epidemiology in areas of relatively lower transmission revealed that acquired immunity also occurred in variable fractions of those populations (Doolan *et al*., 2009). In a longitudinal cohort study of non-immune people recently exposed to endemic malaria, adults required only 2 consecutive acute attacks within 24 months to subsequently become immune to following infections; however, this was not the case with their children (Baird, 1995). Conversely, during the first exposure to acute malaria among migrants, it was adults who were most vulnerable to severe morbidity and mortality relative to their children (Baird *et al*., 1998, 2003; Owusu-Agyei *et al*., 2001). These age- and exposure-dependent patterns seem to hinge on poorly understood age-related host factors. In another study, physiological markers of onset of puberty among African children at variable ages better correlated with onset of protection from febrile illness with patent malaria than age of onset (and cumulative exposure to malaria) (Kurtis *et al*., 2001). Vulnerability to illness with malaria depends on multiple factors, but host age and frequency of recent exposure stand out as dominant.

Populations of wild great apes live with endemic transmission of *Plasmodium* species adapted to them as intermediate hosts, and do so without interventions or known mitigating genetic adaptations. The prevalence of these infections is often quite high (Peters *et al*., 1976; Liu *et al*., 2010, 2017; De Nys *et al*., 2013, 2014, 2017; Wu *et al*., 2018), but very few published studies document the health effects of malaria in these species (Steiper *et al*., 2005; De Nys *et al*., 2017). In the case of orangutans, malaria illness had been reported in rare individual cases (Reid *et al*., 2006), but never studied in detail or at the population level until very recently (Sánchez *et al*., 2022). Utilizing extensive health data longitudinally collected at a Rescue and Rehabilitation Centre (RRC) for orangutans in West Kalimantan, Indonesia, Sánchez et al. (2022) described in detail clinical and parasitological features of endemic natural infection of orangutans caused by *Plasmodium pitheci*, 1 of the 2 species known to naturally infect orangutans. Most of those infections (86%) were asymptomatic, but illness did occur in a minority of cases, sometimes serious and life threatening in character (Sánchez *et al*., 2022).

The study of great apes in the wild comes with conspicuous limitations to access, but some preliminary studies have examined the epidemiology of malaria in great ape species (Wolfe *et al*., 2002; De Nys *et al*., 2013, 2014; Liu *et al*., 2017; Wu *et al*., 2018). Some of these studies reported age as a key factor influencing malaria detection rates for most species of great apes (Wolfe *et al*., 2002; Reid *et al*., 2006; De Nys *et al*., 2013; Mapua *et al*., 2015; Siregar *et al*., 2015). The impact of unmitigated exposure to infection by the plasmodia on all of the great apes is primarily a matter of clinical concern for their conservation, but also of scientific interest as an analogue of human immunity. Epidemiological similarities between the vulnerability of humans and apes to different types of malaria would corroborate the hypothesis for non-sterilizing immunity, which is similar to that in humans (Reid *et al*., 2006; De Nys *et al*., 2013, 2014).

Populations of orangutans living at RRCs within their natural habitats and range expose them to endemic transmission of plasmodia that naturally infect them, *P. pitheci* and *Plasmodium silvaticum* (Peters *et al*., 1976; Wolfe *et al*., 2002; Reid *et al*., 2006; Sánchez *et al*., 2022). These settings provide the means to safely, easily and ethically collect blood samples from wild orangutans under rehabilitation (Wolfe *et al*., 2001; Leendertz *et al*., 2006). The current study was carried out under those circumstances, where orangutan age and exposure to parasitaemia as a determinant of vulnerability to illness with patent infection were examined. An increase in our understanding of this infection will aid in the health management of orangutans living in RRCs before reintroduction into the wild.

## Materials and methods

### Study site and subjects

The *Inisiasi Alam Rehabilitasi Indonesia* Foundation (IAR Indonesia) works under the Directorate of Biodiversity Conservation of the Ministry of Environment and Forestry of the Republic of Indonesia to operate an RRC for Bornean orangutans at Ketapang, West Kalimantan, Indonesia (IAR RRC). This centre began operations in 2009 and has since rescued over 260 orangutans.

### Routine management and health procedures

All orangutans arriving at IAR RRC spend a minimum of 60 days in quarantine. During this time, they undergo medical checks as part of their medical quarantine procedure. Upon completion of the quarantine period, healthy orangutans are transferred to open rehabilitation areas or socialization cages, where they join with conspecifics of approximately the same age and size. Some orangutans are deemed unsuitable for rehabilitation and release, and those become permanent residents of the centre. All other orangutans undergo a rehabilitation process of variable duration that ends with reintroduction into suitable wild protected forested areas of Kalimantan (island of Borneo). The rehabilitation activities take place in semi-natural secondary-forested areas, delimited by artificial canals and electric fences forming islands of free range where they have contact with each other and with wild animals. After successful rehabilitation, orangutans are then transferred and released into a protected natural habitat maintained and designated for this purpose. Intense post-reintroduction monitoring continues for as long as possible to evaluate behaviour and wellbeing.

The health of all orangutans managed at the IAR RRC is closely monitored daily by a team of veterinarians, veterinary assistants and animal keepers. Biological samples are collected only for medical purposes from healthy animals during routine medical check-ups or when orangutans show any signs of illness. Medical records are kept for each orangutan and every medical procedure is recorded both in a hard copy format (paper files) and electronic format (using FileMaker^®^ database).

One of the illnesses diagnosed at this centre is malaria, as reported by Sánchez *et al*. (2022). In this publication, a malaria illness definition has been developed as follows: asymptomatic malaria; clinical (symptomatic) malaria (acute uncomplicated, chronic uncomplicated or mild malaria) and severe malaria (Sánchez *et al*., 2022). Most common clinical malaria symptoms described included: fever and/or lethargy or other general symptoms, accompanied by anaemia and/or thrombocytopaenia and/or leucopaenia.

### Sample collection and classification

As part of routine medical checks conducted on all orangutans at the IAR RRC, blood samples were collected by the staff veterinarians during either manual restraint or incident to clinically indicated anaesthesia. Samples were classified per sampling purpose of each screening event:

1. *Routine medical health checks*: (a) annual health checks; (b) quarantine procedures on arrival and prior to release or (c) health check-ups conducted to monitor the health of the population;
2. *Health monitoring for diagnostic purposes of inpatients* during any illness (except for malaria) and during recumbence periods;
3. *Handling procedures (requiring anaesthesia or not) and minor medical interventions in healthy individuals* for the purpose of transportation, wound cleaning, eye examination and other conditions not considered to affect the general health or the malaria status of the orangutan;
4. *Monitoring the health of patients presenting Plasmodium spp. patent infections*: routine consecutive screening in those individuals known to present patent malaria infection either symptomatically or asymptomatically, for the monitoring of haematology values and/or parasitaemia levels. The finding of plasmodia in blood samples does not prompt chemotherapeutic intervention unless illness consistent with acute or chronic malaria disease is observed, as detailed elsewhere (Sánchez *et al*., 2022).

#### Age category

Age was determined using the individual's dental formula and the age classification as presented in Table 1 and based on descriptions on previous publications (Fooden and Izor, 1983; Smith *et al*., 1994).

**Table 1.** Orangutan age class definition

Age classification	Age	Dentition
Infant (young) (code 1)	4–12 months	First to last deciduous tooth
Infant (old) (code 1)	1–3 years	Last deciduous to first permanent tooth (M1)
Juvenile (code 2)	4–8 years	First permanent tooth to all canines (all permanent teeth except 3rd molars (M3))
Sub-adult (adolescent) (code 3)	9–13 years	At least 1 permanent canine to last permanent tooth (all permanent M3)
Adult (code 4)	14 years on (12–15)	From last permanent tooth (all 4 M3 present) onwards

### Sample analysis

#### Diagnosis of malaria

*Microscopy:* In total, 2105 fresh blood samples were collected by venepuncture and immediately used to prepare thick and thin blood smears on glass slides for microscopic examination to detect the presence of *Plasmodium* spp. If 1 or more sexual or asexual forms of the *Plasmodium* spp. parasite was detected, the sample was classified as positive, while it was classified as negative if after observing at least 100× oil immersion microscopy fields no parasite was detected. All positive blood films contained foreign intracellular microbes having morphologic characteristics compatible with those of plasmodial parasites, and the features of those observed were consistent with a single species, *P. pitheci*, as detailed elsewhere (Sánchez *et al*., 2022).

*Molecular detection by real-time polymerase chain reaction:* Two hundred and thirty-one samples among those tested by microscopy were also analysed using molecular analysis – real-time polymerase chain reaction (PCR). Additionally, 35 samples not examined by microscopy were tested by real-time PCR. The sensitivity of microscopy relative to real-time PCR was estimated to be 79.33% (95% confidence interval (CI) 73.40–85.26). We extracted DNA directly from fresh blood in ethylenediamine tetraacetic acid (EDTA), or from frozen whole blood in EDTA stored at −80 °C using a commercially available kit: PureLink^®^TM Genomic DNA Mini Kit (Invitrogen®TM, ThermoFisher Scientific, Roskilde, Denmark), following the protocol provided by the manufacturer. The extracted DNA was used directly for PCR analysis, or stored at −80 °C. Blood samples were tested by quantitative PCR on a Genesig q16^®^ Real Time-PCR machine (PrimerDesign^®^ Ltd, Eastleigh, UK) using a commercially available kit: *Plasmodium* (all species) Genesig^®^ Easy kit (PrimerDesign^®^ Ltd, UK) following the standard protocol provided by the manufacturer. The primers and probe sequences in this kit, which targets the 18S ribosomal gene, have 100% homology with over 95% of clinically relevant *Plasmodium* spp. references (PrimerDesign-Ltd, 2018). The cut-off cycle threshold value used for this study was 34 cycles (Ct). The lowest parasitaemia load detected below this cut-off value was 2 parasites per μL (>1 parasite μL^−1^).

#### Parasite load in blood

Sexual and asexual parasites in red blood cells were counted by examining Giemsa-stained thick smears by 1000× oil immersion light microscopy until a total of 200 leucocytes (white blood cells (WBCs)) had been observed (Owusu-Agyei *et al*., 2001; Gwamaka *et al*., 2012). To convert the number of parasites observed to a count per microlitre (par μL^−1^) the number observed was multiplied by the actual leucocyte numbers per μL blood and divided by 200 when contemporaneous haematology data were available (Sumbele *et al*., 2015; Raja *et al*., 2020). When those counts were not available, the number observed was simply multiplied by 60 (assuming an average WBC count in orangutans was 12 000 μL^−1^; Sánchez *et al*., 2022). The normal detection limit for parasites in blood using competent microscopy ranges between 4 and 100 par μL^−1^ (Lindblade *et al*., 2014). In this study, the lowest parasitaemia detected by microscopy was 29 par μL^−1^ (Sánchez *et al*., 2022).

### Epidemiology

The prospective epidemiological analysis of malaria in orangutans at the IAR RRC covered a period of active malaria surveillance between 2017 and 2022 with a total of 2140 samples examined by microscopy, PCR (*n* = 35) or both (*n* = 232) from a total of 135 orangutans (62 females and 73 males) observed for an average of 4.3 years or a total of 582.3 orangutan-years.

The initiation of surveillance and follow-up for each individual occurred opportunistically, i.e. when permitted by accessibility of a blood sample for analysis and during random routine tests conducted in the population. Among the 135 orangutans involved, the number of microscopic malaria-independent examinations (i.e. those conducted without regard to malaria infection status) was 1351 in total ranging from 1 to 26 observations per orangutan with an average of 10 per individual. The interval between initial and final observations of blood varied among individuals as newly rescued orangutans were added and others that were removed from the study population during the surveillance period. Immature individuals (infants and juveniles) represented 58% of the time (years) under observation, whereas mature orangutans (sub-adults and adults) represented 42% (Table 2). The minimum observation period for an individual was 5 days and the maximum was 73 months, with an average of 51.8 months of observation time per individual. The results of these examinations were classified as negative, positive asymptomatic or positive symptomatic.

**Table 2.** Number of total samples, total individuals and total orangutan-years at risk for each age category included in this analysis

	No. of samples	No. of individuals	Orangutan-years at risk
Infants	329	43	339 (58.2%)
Juveniles	734	87	
Sub-adults	223	46	243 (41.8%)
Adults	65	24	

### Statistical analysis

#### Incidence rate of clinical malaria (symptomatic malaria infection)

Incidence rate (IR) per 100 orangutan-years of clinical malaria cases (symptomatic parasitaemia) was obtained by reviewing data from inpatient medical records between 2017 and 2022. Inpatient clinical records of all orangutans at the centre are compiled in a computer database (FileMaker^®^). Selection of clinical malaria cases was conducted according to criteria detailed in a previous study (Sánchez *et al*., 2022): a presenting patent malaria infection with over 4000 par μL^−1^ of blood, an increasing density of parasitaemia with an axillary temperature >38 °C, moderate-to-severe normocytic normochromic anaemia (Haemoglobin (HGB) below 8.2 mg dL^−1^), and/or thrombocytopaenia (Platelets (Thrombocytes) lower than 70 × 10^9^ L^−1^) and/or leucopaenia (WBC below 4.7 × 10^9^ L^−1^). General symptoms considered indicative of illness included lethargy defined as any unusual period of inactivity or reduced activity during daily active periods, increased resting activity and reduced responsiveness to stimuli, and/or anorexia defined as decreased appetite. Potentially more severe syndromes involving vital organ impairment included severe neurological signs (coma or mental status impairment, prostration, multiple convulsions and other neurological impairments) in cerebral malaria, kidney failure or respiratory distress (in cases of very acute and severe anaemia).

The IR was defined as the number of new cases of clinical malaria in a year divided by the total orangutan-time at risk (under observation). The IR per year was calculated using an approximate denominator based on the total number of disease-free animals at the start of each year, from which half of the withdrawn animals were subtracted and half of the new additions were added (Dohoo *et al*., 2003). This number was multiplied by 100 to obtain the number of cases per 100 orangutans per year. The IR was calculated for each age category. The overall annual IR was then calculated by summing all the IR for 6 years (2017–2022) and dividing this value by 6.

#### Demographic risk factors for malaria

Age was assessed for risk of parasitaemia and clinical malaria by examining the frequency and density of parasitaemia across age groups. Two-dimensional histograms of parasitaemia levels were generated for each age category across the span of time that had passed since any given individual had first entered the system (usually upon arrival). This allowed us to overlay plots of age groups according to how long individuals within those groups were observed. Shading of points in the histograms (Fig. 2) marked repeated observations. The parasite count was capped at 4000 par μL^−1^ of blood, the threshold for clinical malaria in orangutans (Sánchez *et al*., 2022). These plots reveal the frequency and level of exposure to *P. pitheci* infections of blood.

#### Nested case–control analysis of recent exposure and clinical malaria

Cases included 17 individuals represented by 7 juveniles, 4 sub-adults and 6 adults experiencing an episode of clinical, slide-confirmed malaria during the study period (2017–2022). Thirty-four individuals from different age groups (2 infants, 28 juveniles and 4 sub-adults) having a microscopically confirmed parasitaemia >2000 μL^−1^ without any sign or symptom of illness over the same period, served as controls. Case and control events were limited to otherwise healthy orangutans resident at the RRC for at least 1 year. Records of microscopic examinations of peripheral blood for malaria during the 12 months prior to the defined parasitaemia event were examined for controls, while in the case group, examinations included blood microscopy analysis (*n* = 50) or real-time PCR analysis (*n* = 5) or both (*n* = 2) to determine if at least 1 single malaria positive event (patent or non-patent) had been recorded in the year prior to the clinical case event. Each orangutan was then classified as positive or negative for having experienced at least 1 confirmed parasitaemia in the prior year. An odds ratio (OR) for the absence of prior parasitaemia and clinical malaria was thus calculated.

#### Model

To investigate what influenced the probability of a positive test for *Plasmodium* spp. and in the onset of clinical malaria symptoms when infection was present, a generalized linear mixed-effects model with binomial error structure and logit link function was used (Harrison *et al*., 2018). Age was included as a categorical variable, sex as a binary variable and HGB count as a continuous numerical variable, all modelled as fixed effects, and a random effect intercept for each individual. The age categories used were infant, juvenile, sub-adult and adult status. For the analysis of influence of factors on symptomatic malaria the oldest 2 age groups (mature individuals) were combined as the trend in the analysis for each age group was similar. In this case and also due to the small sample size, a logistic regression without random effects was fit. For comparison of models with and without age information, analysis of variance was used. Due to the small sample sizes, *P* values were not adjusted for multiple comparisons. Statistical analyses were conducted with the R statistical package (R Core Team 2021) and the lme4 module (Bates *et al*., 2015).

## Results

### Age category effects on infection rate of asymptomatic patent infections, parasitaemia levels and incidence of clinical cases

#### Age effect on infection detection rate

Infection detection rates of asymptomatic patent infections (only microscopic examinations) for the different age groups (infants, juveniles, sub-adults and adults) were calculated from the results of 1351 blood samples collected from 132 orangutans over the 6-year period of observation, exclusive of those collected in connection with malaria case management.

Table 3 lists the infection rates in each age category and the corresponding 95% CIs of the estimate. Juveniles had the highest rate of infection at 45.9% (42.3–49.5) followed by infants at 34.7% (29.5–39.8).

**Table 3.** Asymptomatic patent infection rates per age category

	No. of positive samples (no. of total samples)	Infection rate (%)	95% CI
Infants (43 indiv.)	114 (329)	34.7	29.5–39.8
Juveniles (87 indiv.)	337 (734)	45.9	42.3–49.5
Sub-adults (46 indiv.)	59 (223)	26.5	20.7–32.2
Adults (24 indiv.)	15 (65)	23.1	12.8–33.3

To demonstrate whether the difference in infection rates in the different age groups was statistically significant, the data were analysed using a generalized mixed linear model. Table 4 shows the juvenile group was statistically more likely (*P* < 0.005) to have a patent infection compared to the group of mature individuals.

**Table 4.** Age correlation with infection

Term	Estimate	Std. err.	*z*	*P*
Intercept	−1.1214	0.2823	−3.973	7.11 × 10^−5^
Median HGB	−1.0986	0.1675	−6.559	5.43 × 10^−11^
Sex	0.1248	0.2749	0.454	0.64973
Is.infant	−0.1548	0.3183	−0.486	0.62683
Is.juvenile	0.7241	0.2542	2.848	0.00439

#### Age effect on parasitaemia levels

**Figure 1.**
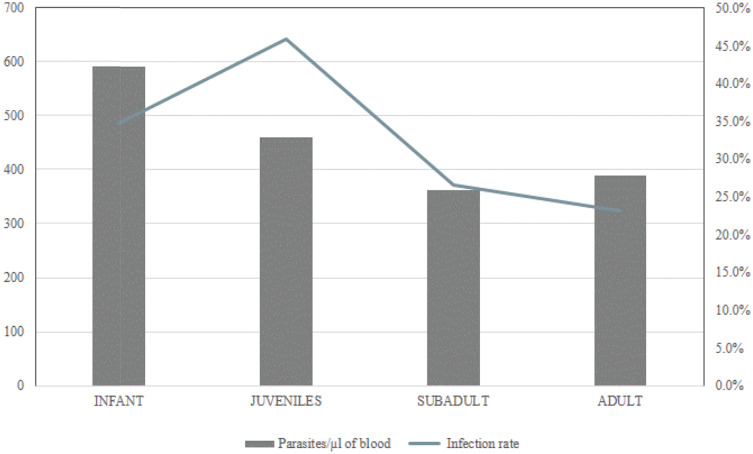
Median of parasites per μL of blood and malaria infection rates in all age groups.

**Figure 2.**
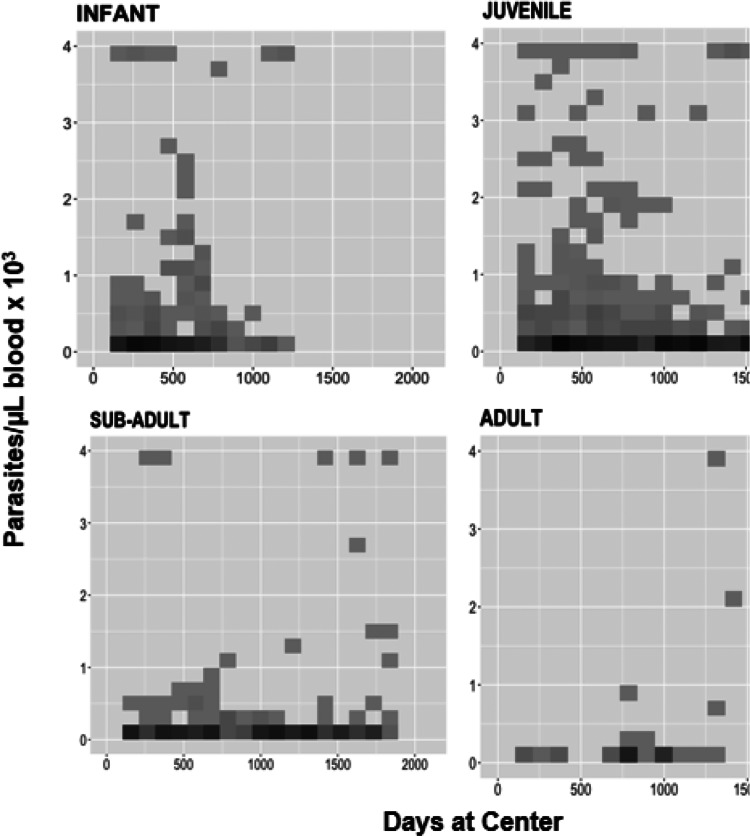
Relatively higher frequencies and levels of parasitaemia among the infant and juveniles (immatures) relative to older orangutans (sub-adults and adults). The immature individuals (top row) consistently showed more frequent and higher density parasitaemia events relative to the mature individuals (bottom row).

Figure 1 illustrates the downward trends in both parasitaemia frequencies and densities as orangutans grow older. Figure 2 illustrates the relatively higher frequencies and levels of parasitaemia among the infant and juveniles (immatures) relative to older orangutans (sub-adults and adults). The immature individuals (top row) consistently showed more frequent and higher density parasitaemia events relative to the mature individuals (bottom row). The highest median parasitaemia levels occurred in infants (592 par μL^−1^), and the lowest was in sub-adults (362 par μL^−1^).

#### Age effect on IR of clinical malaria

We calculated the IR of symptomatic malaria (clinical malaria cases). The IR of clinical malaria (symptomatic malaria) is defined as the number of clinical malaria attacks per 100 individuals per year. The data of 17 clinical cases recorded between 2017 and 2022 were used, excluding recurrent episodes in 2 of the individuals. Table 5 lists the annual IR for each age category.

**Table 5.** Clinical malarial IR in the total population and per age in a period between 2017 and 2022 in 17 clinical malaria cases (excluding 3 recurrent episodes in 2 of the orangutans)

	Annual IR (events/100 orangutans)
Infants	0.0
Juveniles	2.1
Sub-adults	5.2
Adults	6.0

The highest IR was recorded in the adult group (6.0 cases per 100 orangutan-years) followed by the sub-adult group (5.2 cases/100 orangutan-years) and the juvenile group (2.1 cases/100 orangutan-years). No clinical malaria was recorded among infants over the study period. Relative to juveniles, adults were nearly 3 times more likely to experience clinical malaria.

A generalized linear mixed-effects model was used in order to determine whether the onset of clinical malaria was statistically associated with age (Table 6). The results showed that the group of mature individuals (sub-adult/adults) was statistically more likely (*P* < 0.01) to suffer from clinical malaria (symptomatic patent infection) compared to the juvenile group (Table 6).

**Table 6.** Generalized linear mixed-effects model results of clinical malaria (*n* = 20 cases; including 3 recurrent episodes in 2 of the orangutans) and age

Term	Estimate	Std. err.	*z*	*P*
Intercept	−6.4341	1.0730	−5.996	2.02 × 10^−9^
Median HGB	−2.9565	0.5621	−5.260	1.44 × 10^−7^
Sex	−0.6474	0.7253	−0.893	0.372
Is.adult/sub-adult	2.0227	0.7500	2.697	0.007

### Case–control analysis of recent exposure to infection and clinical malaria

Table 7 lists the numbers of symptomatic cases having at least 1 episode of patent parasitaemia in the year prior to that event *vs* the same in individuals experiencing an asymptomatic parasitaemia event. Orangutans experiencing clinical malaria were over 259 times more likely to have not experienced an episode of patent malaria in the year leading to the attack relative to orangutans experiencing an asymptomatic episode of patent parasitaemia.

**Table 7.** OR of exposure to patent parasitaemia in the previous 52 weeks in individuals that had suffered a clinical malaria case (cases) and in individuals that had experienced asymptomatic parasitaemic events of >2000 par μL^−1^ in the absence of malaria symptoms (controls)

Patent parasitaemia event	Parasitaemia during prior 12 months		OR (95% CI; *P* value)
	No	Yes	
Clinical case	16	1	256 (21.56–3039.83; <0.0001)
Asymptomatic control	2	32	

**Figure 3.**
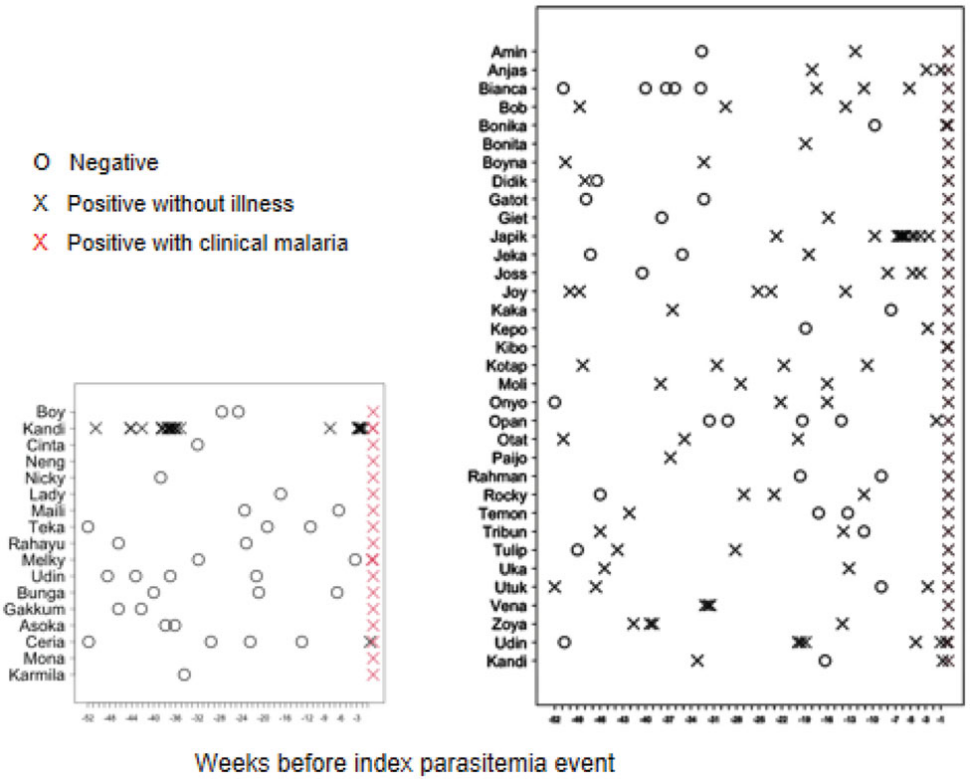
Left: Microscopic surveillance among 17 clinical malaria cases (symptomatic) that formed the group of cases during 52 weeks prior to the clinical illness event. The only case where previous asymptomatic infection had been detected (Kandi) was recorded as a mild malaria case not requiring medical treatment. Right: Microscopic surveillance among 34 asymptomatic cases that formed the group of controls during 52 weeks leading to the event of high parasitaemia (>2000 par μL^−1^) in the absence of malaria symptoms.

Figure 3 illustrates the numbers and timing of blood film examinations and findings for both cases and controls. Among cases, there were a total of 59 microscopic examinations with just 1 of 17 of those individuals being positive in the 12 months period before the recorded symptomatic event. Fifteen cases had at least 1 examination and for 2 of them there was no examination in the previous year. The median number of examinations among cases was 2. Among controls, a total of 121 examinations performed, with 32 of 34 having at least 1 positive examination in the previous year. All 34 controls had at least 1 examination, and the median number of examinations among them was 3.

## Discussion

The parasitological and clinical observations reported here are in accord with the onset of naturally acquired immunity to *P. pitheci* malaria in Bornean orangutans in an age- and exposure-dependent manner. Infant and juvenile orangutans presented higher infection rates while juveniles also presented a higher probability of positive asymptomatic infection (*P* < 0.005) (Table 4). Median parasitaemia level in asymptomatic infections was the highest in the infant group and it decreased with age (Fig. 1). Immature orangutans experienced more frequent and higher-grade parasitaemia events compared to the mature individuals (Fig. 2). The occurrence of lower-grade and less-frequent parasitaemia events among mature orangutans suggests suppression of infection by immunity acquired over periods of nearly continuous exposure.

Paradoxically, the mature orangutans proved more susceptible to the onset of clinical illness with *P. pitheci* infection (Table 5) with the group of mature individuals (sub-adult/adults) statistically more likely (*P* < 0.01) to suffer symptomatic patent infection compared to the juvenile group (Table 6). Similar seemingly discordant findings have been obtained in human malaria; malaria-naïve adults exposed to endemic risk have been consistently more susceptible to poor clinical outcomes compared to younger age groups (Baird, 1998; Baird *et al*., 2003, 2012; Basri *et al*., 2003; Doolan *et al*., 2009). Among the non-immune, the young are less vulnerable to serious illness. We tested the hypothesis that the relative susceptibility to illness with *P. pitheci* parasitaemia observed in mature orangutans could have been caused by a period of prolonged absence of exposure to parasitaemia, as is known to occur in human *Plasmodium falciparum* malaria (Doolan *et al*., 2009; Guinovart *et al*., 2012; Kinyanjui, 2012; Lindblade *et al*., 2014). Our findings in the case–control study supported that hypothesis, i.e. the orangutans experiencing symptomatic malaria (cases) were over 200 times more likely to have been free of observed parasitaemia during the year leading to illness compared to orangutans experiencing a relatively heavy (>2000 par μL^−1^) but asymptomatic parasitaemia (Table 7). This apparent lack of recent exposure to parasitaemia, likely occurring by chance in the varied habitats of the RRC, seems to have resulted in a waning of protection from higher parasitaemia events and acute illness with *P. pitheci* malaria. This epidemiology of malaria illness resembles that occurring in settings of meso-endemic human malaria where most adults harbour asymptomatic parasitaemia events but a minority progress to severe and threatening malaria (Doolan *et al*., 2009; Lindblade *et al*., 2014). Orangutans exposed to parasitaemia by *P. pitheci* exhibit protection from associated illness, whereas those lacking similar exposure appear vulnerable to disease. Naturally acquired immunity to *P. pitheci* malaria thus resembles that occurring with *P. falciparum* malaria in human populations.

The potential pitfall of an apparent lack of exposure being the result of a lack of observations among the ill cases relative to asymptomatic controls was addressed. The observations illustrated in Fig. 3 show that the number and frequency of observations was similar between cases and controls. A relative lack of years at risk among immature orangutans constituted another potential bias creating an illusion of protective immunity in them. However, immature orangutans contributed over 55% of the total 579 orangutan-years under observation (Table 2).

Age is widely considered a factor influencing outcomes of malaria infection in humans as well as in great apes. Two previous orangutan malaria studies also reported a higher prevalence of *Plasmodium* spp. infection in younger *vs* older orangutans (Wolfe *et al*., 2002; Reid *et al*., 2006) suggesting that age could be an epidemiologic factor influencing malaria infection status. Density of parasites in blood is a factor affecting morbidity and mortality in human malaria (Doolan *et al*., 2009). In orangutan malaria disease, higher parasitaemia levels in blood have also been correlated with clinical outcomes with clinical malaria cases reported to be above approximately 3000–4000 par μL^−1^ in 1 study (Sánchez *et al*., 2022).

While immature orangutans experienced a high rate of asymptomatic infections and their parasitaemic levels were higher than those in the older groups, the probability of suffering from clinical malaria was lower in the former group. No case was detected in any infant orangutan during the surveillance period (2017–2022). However, in human malaria, infants and small children are more susceptible to severe illness in endemic areas (Baird *et al*., 1998, 2003; Doolan *et al*., 2009; Guinovart *et al*., 2012; World Health Organization, 2016, 2019). Only 1 severe malaria case had been reported at this RRC years prior to this study (in 2011). This severe case involved an infant orangutan (approximately 2 years) which presented cerebral malaria (Sánchez *et al*., 2022). Malaria severity and mortality although unlikely, is possible, and potentially with even more severe effects in the infants *vs* in the older individuals, especially in the absence of appropriate treatment (Sánchez *et al*., 2022).

Accounting for age as a factor influencing malaria detection rates might also be important in epidemiology studies in captive *vs* wild populations. Previous studies reported higher infection rates in rehabilitant *vs* wild orangutans (Wolfe *et al*., 2002) while another study detected a higher proportion of infections in infant wild orangutans rescued from captivity *vs* rehabilitants (Reid *et al*., 2006). It was assumed that rehabilitant orangutans and especially those among humans would be more at risk of suffering from plasmodial infections. However, the first study had a larger sample size of adults, while the second study had a larger proportion of infants. Those results were perhaps confounded by the age of the individuals sampled.

Several reports on orangutan RRCs have indicated a high incidence of clinical malaria cases in orangutans undergoing rehabilitation or at the time of reintroduction into the wild (Sánchez *et al*., 2022) which simultaneously also suffer from increased stress and cortisol levels (Reid *et al*., 2006; Russon, 2009). An increased cortisol level is believed to be a factor increasing the risk of malaria disease in other animal species as well as in humans (Doolan *et al*., 2009; Names *et al*., 2021). However, a study conducted on gorillas did not find any correlation between high stress levels and malaria detection rates (Mapua *et al*., 2015). Furthermore, no stress factors were believed to affect this cohort of orangutans during this study.

A more plausible scenario to explain the high incidence of clinical malaria cases is an inadequate immune response to patent infection by the host; a history of prolonged interruption of exposure to infection in rehabilitant orangutans may result in an increased susceptibility to more severe effects of malaria illness when coming into contact with malaria vectors – e.g. after reintroduction into the wild – negatively impacting the health and hence the welfare and even the survival of these individuals. While the IUCN Guideline for Reintroduction of Great Apes emphasizes the importance of ensuring the health of individuals released into the wild to protect the health of wild populations (Beck *et al*., 2007; Russon, 2009) very little is mentioned about the reverse effect, the potential transmission of pathogens from wild populations to the reintroduced apes.

Mosquito species thrive within specific ecological conditions and as such, species found in forested areas might be different from those found in non-forested areas and urban sites (Afrane *et al*., 2008; Hawkes *et al*., 2019). Although vector species that transmit malaria in orangutans have not been identified yet, in other great ape species, mosquito vectors are considered to be strictly forest species (Scully *et al*., 2022). It is widely documented that anthropogenic changes in environmental conditions, such as land-use changes, can alter the ecology of vectors and hence how they breed, develop and transmit disease, hence affecting malaria epidemiology in humans (Chang *et al*., 1997; Afrane *et al*., 2008; Jiram *et al*., 2012; Moyes *et al*., 2014, 2016; Brant *et al*., 2016; Austin *et al*., 2017; Brown *et al*., 2018, 2019; Hawkes *et al*., 2019). Deforestation and forest fragmentation force orangutans to live in altered landscapes affected by ecological changes (e.g. forest-edge habitats, in and around agricultural areas and/or human settlements) in which vector mosquitos might be absent or their capacity to transmit malaria inhibited. This might result in an impairment of immunity and an increased risk of more serious health implications of malaria infection in these populations. Thus, malaria disease might represent a new threat to the conservation of orangutan populations living in anthropogenic and forest-fragmented landscapes. The identification of vectors involved in the transmission of malaria and how they are affected by ecological changes will be essential in understanding orangutan malaria epidemiology and in developing measures for its management and prevention.

Infectious diseases can have deleterious effects with potentially serious implications for the conservation of already threatened great ape populations, and are increasingly considered a threat to the survival of wildlife species (Wolfe *et al*., 1998; Leendertz *et al*., 2006; Machalaba *et al*., 2020). Increasing our understanding of diseases affecting wild orangutans is of paramount importance not only for the wellbeing and survival of the species (*One Welfare*) but also for its contribution to public health security and disease control (*One Health*).

## Data Availability

The data that support the findings of this study are available on request from the corresponding author, [KLS]. The data are not publicly available due to government ownership.
